# Analysis of the human immunodeficiency virus type 1 M group Vpu domains involved in antagonizing tetherin

**DOI:** 10.1099/vir.0.035931-0

**Published:** 2011-12

**Authors:** Sarah J. Petit, Caroline Blondeau, Greg J. Towers

**Affiliations:** MRC Centre for Medical Molecular Virology, Division of Infection and Immunity, University College London, Cruciform Building, 90 Gower Street, London WC1E 6BT, UK

## Abstract

Zoonosis of chimpanzee simian immunodeficiency virus cpz to humans has given rise to both pandemic (M) and non-pandemic (O, N and P) groups of human immunodeficiency virus type-1 (HIV). These lentiviruses encode accessory proteins, including Vpu, which has been shown to reduce CD4 levels on the cell surface, as well as increase virion release from the cell by antagonizing tetherin (CD317, BST2). Here, we confirm that O group Vpus (Ca9 and BCF06) are unable to counteract tetherin or downregulate the protein from the cell surface, although they are still able to reduce cell-surface CD4 levels. We hypothesize that this inability to antagonize tetherin may have contributed to O group viruses failing to achieve pandemic levels of human-to-human transmission. Characterization of chimeric O/M group Vpus and Vpu mutants demonstrate that the Vpu–tetherin interaction is complex, involving several domains. We identify specific residues within the transmembrane proximal region that, along with the transmembrane domain, are crucial for tetherin counteraction and enhanced virion release. We have also shown that the critical domains are responsible for the localization of M group Vpu to the *trans*-Golgi network, where it relocalizes tetherin to counteract its function. This work sheds light on the acquisition of anti-tetherin activity and the molecular details of pandemic HIV infection in humans.

## Introduction

Zoonosis of chimpanzee simian immunodeficiency virus (SIVcpz) to humans has given rise to both pandemic (M) and non-pandemic (O, N and P) groups of human immunodeficiency virus type-1 (HIV). Tetherin (BST-2, CD317) is an interferon-inducible protein that restricts the release of enveloped viruses by tethering the newly budded virions to the plasma membrane and causing their endocytosis back into the cell for destruction (Neil *et al.*, 2008). Tetherin is a single pass type II membrane protein containing a C-terminal GPI anchor that localizes mainly to the cell membrane and the *trans*-Golgi network (TGN) (Neil *et al.*, 2008). Lentiviruses have evolved various anti-tetherin activities mediated by viral proteins including Vpu, Nef and the envelope protein (SIVmac/tan/HIV-2) (Gupta *et al.*, 2009b; Hauser *et al.*, 2010; Jia *et al.*, 2009; Le Tortorec & Neil, 2009; Martin-Serrano & Neil, 2011; Neil *et al.*, 2008; Serra-Moreno *et al.*, 2011; Van Damme *et al.*, 2008; Zhang *et al.*, 2009). Vpu is a small accessory protein with roles in CD4 cell surface downregulation and tetherin antagonism (Klimkait *et al.*, 1990; Willey *et al.*, 1992). Vpu recruits the cytoplasmic tail of CD4 in the endoplasmic reticulum (ER) thereby preventing it from recruiting the viral envelope protein gp160 and sequestering from the cell surface (Malim & Emerman, 2008). It simultaneously binds CD4 and β-TrCP via its DSGXXS motif, which recruits SCF (Skp1/Cullin/F-box protein) E3 ubiquitin ligase, prompting ubiquitination and CD4 degradation in the proteasome (Margottin *et al.*, 1998). Vpu has also been shown to remove tetherin from the cell surface (Skasko *et al.*, 2011; Tokarev *et al.*, 2011; Van Damme *et al.*, 2008) by a β-TrCP-dependent mechanism (Mangeat *et al.*, 2009). Removal of tetherin from the cell surface is not essential for antagonism as Vpu–tetherin interaction was sufficient for a partial rescue of virion release (Mangeat *et al.*, 2009). This presumably means that tetherin–Vpu complexes are unable to tether virions even if they are at the cell surface.

HIV-1 NL4-3 M group Vpu has species-specific anti-tetherin activity against human but not simian tetherins (Goffinet *et al.*, 2009; Gupta *et al.*, 2009a; McNatt *et al.*, 2009). Furthermore, HIV-1 M group Vpus antagonize tetherin from apes and humans but not monkeys. Moreover, exchanging the tetherin transmembrane domain between human tetherin and African green monkey or rhesus macaque tetherins exchanged sensitivity to Vpu (Jia *et al.*, 2009; Lim *et al.*, 2010; Sauter *et al.*, 2009). SIVcpz Vpus failed to antagonize tetherins from a variety of hosts, including chimpanzees, and SIVgor had a very restricted anti-tetherin activity (Sauter *et al.*, 2009). As SIVcpz uses Nef to antagonize chimpanzee tetherin it has been suggested that HIV-1 gained the ability to antagonize tetherin using Vpu, rather than Nef, as a part of its adaptation to its new human host (Gupta & Towers, 2009; Sauter *et al.*, 2009; Zhang *et al.*, 2009). Intriguingly, three independent O group Vpus had no activity against human tetherin, suggesting that the O group viruses have failed to make this adaptation (Sauter *et al.*, 2009). In order to try to understand the molecular adaptation of group M versus the failure of group O Vpu to antagonize tetherin, we undertook a detailed mutational analysis of Vpu function. Chimeras and mutants of M and O group Vpus allowed us to identify specific domains within the M group sequence that are responsible for Vpu’s acquired anti-tetherin function.

## Results

### Analysis of Vpu domains required to antagonize tetherin

**Fig. 1.**
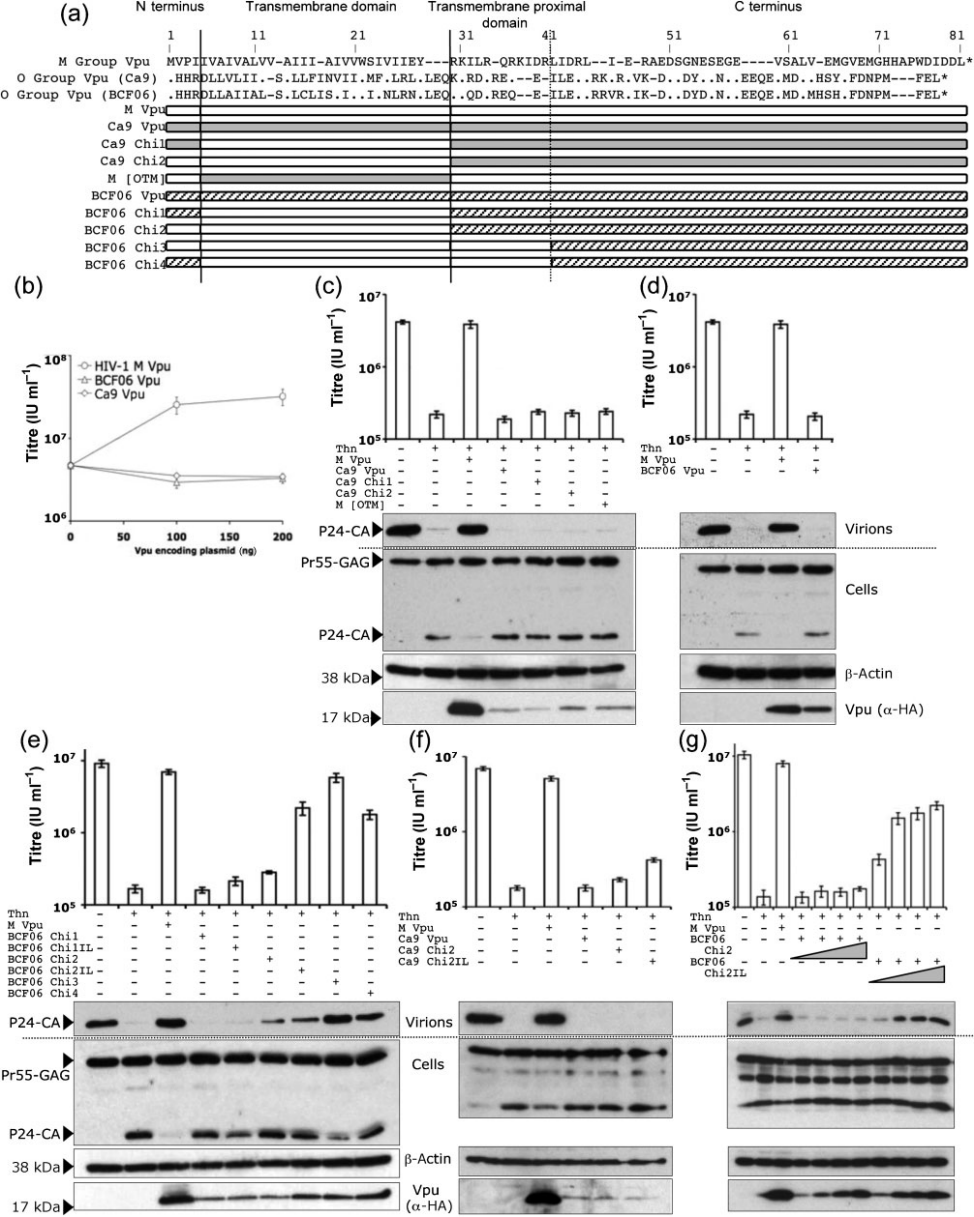
M group Vpu has evolved to antagonize tetherin through its TM and TM proximal domains. (a) Alignment of NL4-3 M group and Ca9 and BCF06 O group Vpus, as well as schematics of the Vpu chimeras. (b) Titres of HIV-1 released from 293T cells cotransfected with a titration of NL4-3, Ca9 or BCF06 Vpu encoding plasmids, HIV-1 vectors and 100 ng human tetherin. (c, d, e, f and g) Titres of HIV-1 released from 293T cells cotransfected with 500 ng [(g) 200, 500, 700 and 1000 ng] of various Vpu encoding plasmids, HIV-1 vectors and 100 ng human tetherin. P24 staining of Western blots of viral supernatants and cell lysates reflect viral titres and indicate equal Gag expression. Vpu expression was measured by detecting the C-terminal HA-tag and β-actin was measured for loading control. Results are representative of three separate experiments.

HIV-1 has entered the human population through at least four different zoonotic events, leading to HIV-1 viruses: pandemic HIV-1 M and non-pandemic groups N, O and P. Previous studies have shown that Vpus from M group viruses are able to antagonize human tetherin, whereas O group Vpus are not (Sauter *et al.*, 2009). Furthermore, Vpu sequences are very variable (Fig. 1a). We confirmed M group Vpu antagonism of tetherin, as well as the lack of tetherin antagonism by previously untested O group Vpus from HIV-1s Ca9 and BCF06 (Janssens *et al.*, 1999; Loussert-Ajaka *et al.*, 1995). Vpus were titrated against a fixed amount of human tetherin plasmid in transient transfection assays in 293T cells and their ability to antagonize human tetherin tested as described previously (Gupta *et al.*, 2009b). Expression of human tetherin strongly inhibited the release of HIV-1 encoding YFP as described previously (Neil *et al.*, 2008; Sauter *et al.*, 2009), and NL4-3 M group Vpu expression, but not O group Vpus Ca9 or BCF06, were able to rescue HIV-1 YFP release (Fig. 1b). The Vpu proteins were untagged, precluding measurement of Vpu protein levels. To consider which domains of M group Vpu have successfully adapted to antagonize tetherin, we prepared C-terminally HA-tagged chimeras between active NL4-3 M group Vpu and the inactive O group Vpus (Fig. 1a). As Vpu was previously shown to bind tetherin through its transmembrane domain (Iwabu *et al.*, 2009), we swapped the transmembrane domain of Ca9 and BCF06 Vpu with that from NL4-3 Vpu and assayed anti-tetherin function (Fig. 1c–e). Full-length M group NL4-3 Vpu completely rescued virus release as expected, whereas a chimeric NL4-3 Vpu with the Ca9 O group transmembrane region, M [OTM], did not (Fig. 1c). This supported the notion that the Ca9 transmembrane domain was defective in its ability to antagonize tetherin function. However, the opposite chimera of the O Group Ca9 Vpu with the NL4-3 transmembrane domain (Ca9 Chi1) was also unable to enhance virion release (Fig. 1c). Adding the N-terminal tail of the M group Vpu to the Ca9 chimera such that it encoded M Vpu residues 1–29 (Ca9 Chi2), still did not allow it to antagonize tetherin. Relative HIV-1 p24 levels in viral supernatants reflected relative viral titres as expected, and cell lysate levels of HIV-1 proteins were similar (Fig. 1c). However, analysis of the HA-tagged Vpu expression levels revealed that the Ca9 O group Vpu and chimeric Vpus were poorly expressed, suggesting an alternative reason for their lack of activity (Fig. 1c). However, titration of Ca9 and its chimeras revealed that expression levels could not account for Ca9’s inactivity (Supplementary Fig. S1, available in JGV Online). Nonetheless, we tested another, better expressed, Vpu from O group HIV-1 BCF06 (Loussert-Ajaka *et al.*, 1995). This was expressed almost as well as HA-tagged NL4-3 Vpu and was unable to antagonize tetherin (Fig. 1d). Furthermore, BCF06 chimeras 1 and 2 were unable to antagonize tetherin (Fig. 1e) although their expression was higher than that of the Ca9 chimeras (Fig. 1e). We conclude that the transmembrane domain of NL4-3 M group Vpu is necessary for tetherin antagonism but is insufficient to confer tetherin antagonism to O group Vpus, even in the presence of the M group N terminus.

Inspection of Vpu sequences revealed that the M group transmembrane proximal domain includes a positively charged region including an isoleucine leucine (IL) aliphatic motif, which constitutes a YXXφ motif, whereas O group sequences contain charged residues at these positions. To test whether this motif is important for tetherin antagonism we added the membrane proximal region of NL4-3 Vpu to BCF06 chimera 2 to make BCF06 chimera 3 (Fig. 1a, e). BCF06 chimera 3, which encodes M Vpu residues 1–40, was able to enhance virion release, exhibiting anti-tetherin activity almost as effective as NL4-3 Vpu (Fig. 1e). Replacing the small N-terminal tail of BCF06 chimera 3 with that of O group (BCF06 Chi4) slightly reduced the ability of the chimera to enhance virion release, suggesting that the N terminus of the M group Vpu also has a role in anti-tetherin function. Supernatant titres reflected supernatant p24 levels confirming antagonism of tethering. As the OTM chimera cannot antagonize tetherin we conclude that both the transmembrane proximal domain and the transmembrane domain of NL4-3 Vpu are essential for conferring anti-human tetherin activity to HIV-1 M group Vpu.

To further examine the role of the I32, L33 aliphatic motif in tetherin antagonism we took the BCF06 Chi1 and 2, which bear the M group transmembrane region, and mutated residues Q_32_D_33_ to IL, their equivalents in the M group sequence. BCF06 Chi1 Q_32_I, D_33_L (BCF06 Chi1IL) did not gain anti-tetherin function but BCF06 chimera 2 Q_32_I, D_33_L (BCF06 Chi2IL), which additionally includes the M group N-terminal domain, was able to rescue HIV-1 release to levels slightly below the M group Vpu (Fig. 1e). Analysis of p24 supernatants confirmed tetherin antagonism. These results indicate that the N terminus, the isoleucine and leucine residues in the membrane proximal domain, as well as the transmembrane domain, are required for BCF06 Vpu to gain anti-tetherin activity. Finally, we mutated Ca9 chimera 2 Vpu to include the same membrane proximal I32, L33 motif (Fig. 1f). Ca9 chimera 2 R_32_I, D_33_L (Ca9 Chi2IL) remained unable to rescue viral release effectively, even at high levels of expression, although a weak enhancement was evident on addition of the IL motif. Importantly, the titration demonstrated that inactivity is not due to poor expression as the high levels of Ca9 Chi2IL are less effective than low amounts of M group Vpu (Supplementary Fig. S1).

To further confirm that poor expression levels were not responsible for the inability to antagonize tetherin function by the various Vpu constructs, we performed a titration of four increasing doses of the functional chimera BCF06 Chi2IL and its non-functional counterpart BCF06 Chi2 (Fig. 1g). As above, BCF06 Chi2 was unable to enhance virion release even at expression levels that were higher than BCF06 Chi2IL, which exhibited potent anti-tetherin activity at lower doses. A lack of anti-tetherin activity is thus not due to poor expression levels, but due to the protein sequence. As a further control, we also titrated the other constructs showing that Ca9, BCF06, Ca9 Chi2, Ca9 Chi2IL and BCF06 Chi1IL also have no activity despite being expressed at levels equal to or higher than the functional M group Vpu (Supplementary Fig. S1).

### Analysis of domain requirements for removal of CD4 and tetherin from the cell surface

**Fig. 2.**
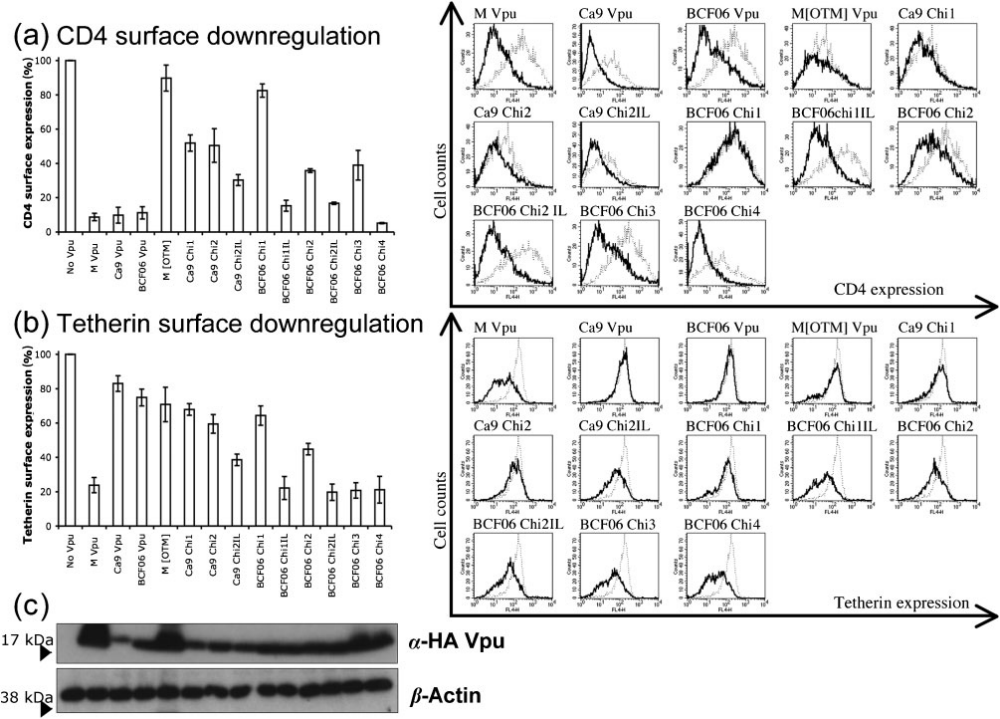
M and O group Vpus downregulate CD4 and tetherin differently from the cell surface. (a) 293T cells were cotransfected with a CD4 expression vector and pIRES2eGFP plasmids expressing GFP alone (no Vpu) or together with the indicated Vpu constructs, and CD4 surface expression was measured by FACS. Shown are relative levels of CD4 cell surface expression (black line) relative to those measured in cells transfected with the GFP only control vector (dotted line). (b) 293T cells were cotransfected with an ectodomain HA-tagged human tetherin vector and pIRES2eGFP plasmids expressing GFP alone (no Vpu) or together with the indicated Vpu constructs and HA tetherin surface expression was measured by FACS. Shown are the relative levels of tetherin cell surface expression (black line) relative to those measured in cells transfected with the GFP only control vector (dotted line). Results are mean±sem of three separate experiments. (c) Western blot of HA-tagged Vpu in the pIRES contruct showing expression of the various Vpu constructs, as well as a β-actin control.

An important function of HIV-1 Vpu is to reduce cell surface CD4 levels (Margottin *et al.*, 1998; Willey *et al.*, 1992). To test whether the O group or the chimeric Vpus reduce surface expression of CD4 similarly, 293T cells were co-transfected with plasmids encoding Vpu and a plasmid encoding CD4. CD4 surface levels were then measured by flow cytometry. The results examining Vpu mediated removal of CD4 from the cell surface were surprisingly complex, particularly given that both M group and O group Vpus strongly reduced surface CD4 levels (Fig. 2a) as described previously (Sauter *et al.*, 2009). Even the poorly expressed Ca9 O group Vpu was able to effectively reduce surface CD4. Surprisingly, the M group Vpu with an O group transmembrane domain and the opposite chimera (BCF06 chimera 1, with an NL4-3 transmembrane domain) were completely inactive in this assay. Most of the other chimeras were somewhat active, reducing CD4 by 40–60 % of control levels. Notably, the membrane proximal region was important for CD4 downregulation as well as anti-tetherin activity (Fig. 1). For example, BCF06 chimera 1 was unable to remove CD4 from the membrane but this chimera mutated to include I32, L33 had wild-type activity. This was the most striking example of a role for I32, L33 but the incorporation of this motif also improved the ability of all the other Vpu chimeras tested, against CD4. A complex role for the Vpu N terminus is also revealed by these experiments. The addition of the M group N terminus to BCF06 chimera 1 to make chimera 2 improves anti-CD4 activity, yet adding the N terminus to chimera 4, which additionally bears the M group membrane proximal region, to make chimera 3 reduces anti-CD4 activity. It therefore appears that the membrane proximal region dictates the importance of the N terminus, which is distant from it and on the other side of the plasma membrane. We conclude that the Vpu protein is a compact and complex molecule in which, perhaps not surprisingly, it is very difficult to separate its functions into domains. Our observations suggest that different Vpu proteins, particularly when comparing M and O Vpus, use different combinations of parts of Vpu to achieve the same effect on a host molecule, in this case CD4.

**Fig. 5.**
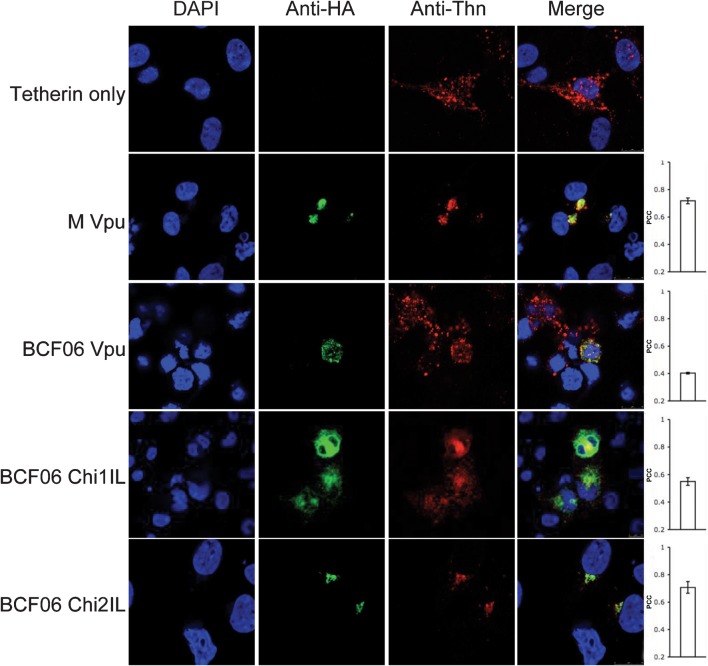
O group Vpu does not relocalize tetherin to the TGN. Confocal images of 293T cells cotransfected with the various Vpu constructs and human tetherin and stained with immunofluorescently labelled anti-HA (Alexa-488) and anti-tetherin (Alexa-594) antibodies. Cell nucleus is detected with DAPI and merged images are in the fourth column. Each image is representative of at least four different fields positive for each HA-construct, taken at random. The PCC was calculated for each HA-construct. Values represent mean±sem.

Vpu has also been shown to reduce cell surface tetherin levels (Gupta *et al.*, 2009b; Neil *et al.*, 2008; Sauter *et al.*, 2009; Van Damme *et al.*, 2008). We therefore tested our Vpu constructs for their ability to reduce cell surface tetherin levels. We used a flow cytometry assay, as above (Fig. 2b). NL4-3 M group Vpu reduced surface tetherin signal by 75 %, whereas the O group Vpus only reduced it by about 20 %. The chimeras lacking anti-tetherin activity namely Ca9 chimeras 1 and 2, BCF06 chimeras 1, and M [OTM] were all unable to efficiently remove tetherin from the cell surface, leaving 60–70 % of the surface tetherin signal. Likewise, Ca9 chimera 2 I32, L33 and BCF06 chimera 2, which were all defective in antagonizing tetherin in a virion release assay (Fig. 1), were unable to reduce tetherin levels beyond 50–60 % of control. Surprisingly, BCF06 chimera 1 with the addition of the I32, L33 motif was able to reduce cell surface tetherin levels to 20 %, the same as wild-type NL4-3 Vpu, but it was unable to rescue virion release (Fig. 1). This anomaly is examined further, measuring tetherin localization, see Fig. 5. Importantly, all of the Vpu chimeras that were functional in antagonizing tetherin and rescuing viral particle release (BCF06 chimeras 2IL, 3 and 4) were also able to efficiently reduce the tetherin signal to 20 % of the control, as was M group Vpu.

In order to verify that the downregulation activity observed for these Vpu constructs was not due to their level of expression, these experiments were repeated with HA-tagged Vpu pIRES constructs. The same pattern as the untagged constructs was observed, although the degree of downregulation was slightly less (data not shown). Vpu expression levels were also tested by Western blot, detecting HA-tagged Vpu pIRES constructs (Fig. 2c). The Vpus showed the same expression pattern as for the pcDNA3 constructs in Fig. 1. CD4 and tetherin downregulation from the cell surface were thus not a function of Vpu expression levels.

### Localization of M and O group Vpus reflects their anti-tetherin activity

**Fig. 3.**
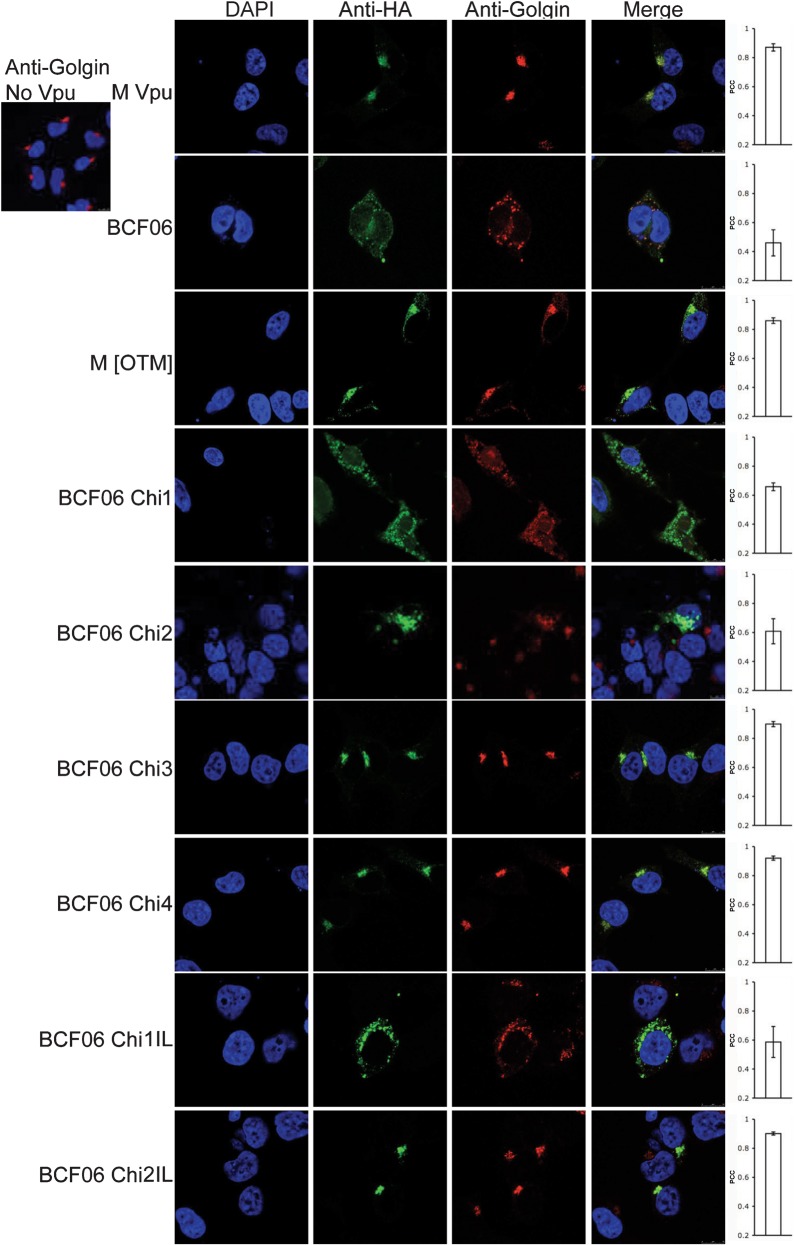
M and O group Vpus’ localization reflect their anti-tetherin activity. Confocal images of 293T cells transfected with the various Vpu constructs and stained with immunofluorescently labelled anti-HA (Alexa-488) and anti-p230 *trans*-Golgi (anti-Golgin, rhodamine). Cell nucleus is detected with DAPI and merged images are in the fourth column. Each image is representative of at least four different fields positive for each HA-construct, taken at random. The PCC was calculated for each HA-construct. Values represent mean±sem.

Vpu localization is important for its anti-tetherin function (Dubé *et al.*, 2009; Hauser *et al.*, 2010). We therefore examined the intracellular location of M and O group Vpu using immunofluorescence, as described previously (Gupta *et al.*, 2009b). We found a correlation between anti-tetherin activity and association with the TGN. M group NL4-3 Vpu is co-localized with Golgin, suggesting a TGN location, as described previously (Dubé *et al.*, 2009; Hauser *et al.*, 2010) (Fig. 3). Colocalization was confirmed by quantification and measurement of Pearson’s correlation coefficient (PCC; M group Vpu PCC = 0.87) (Dunn *et al.*, 2011; Manders *et al.*, 1993). However, O group Vpu from BCF06 is found in a diffused vesicular pattern and on the cell membrane (Fig. 3) (BCF06 Vpu PCC = 0.46). The Vpu chimeras that could not antagonize tetherin; namely chimera 1 (PCC = 0.66) with the transmembrane domain of NL4-3 and chimera 2 (PCC = 0.61), that additionally has the NL4-3 N-terminal domain, have a diffuse cellular localization, similar to that of wild-type BCF06 Vpu. Alternatively, the active chimera 3, which bears residues 1–39 of NL4-3 Vpu, including the membrane proximal region, shows the same punctate *trans*-Golgi localization as wild-type NL4-3 Vpu (PCC = 0.9) (Fig. 3). The same pattern occurs for the active chimera 4 (PCC = 0.92), which has the O group N terminus, rather than that from NL4-3. Chimera 1IL (PCC = 0.59), which has the I32, L33 motif does not antagonize tetherin and is not found in the punctate TGN localization, but dispersed throughout. Thus, the IL motif on its own does not rescue chimera 1’s localization or activity. However, the addition of the NL4-3 N terminus to this chimera (1IL) to make chimera 2IL (PCC = 0.9), redirects it to the punctate TGN location and rescues virus release Fig. 1(e). Thus, control of localization to the TGN and tetherin antagonism appear to be complex and involve both the N terminus and the membrane proximal region. The N terminus is only required in chimeras that encode just I and L at positions 32–33, but not the rest of the M group membrane proximal region. In other words redundancy between residues in the membrane proximal region and the N terminus for TGN association is suggested by the observation that chimera 4, which lacks the M group N terminus but includes the entire M group membrane proximal region, is also localized to the TGN.

The transmembrane swap [OTM], comprising NL4-3 Vpu with an O transmembrane, localized punctately to the TGN (PCC = 0.86) (Fig. 3), but could not antagonize tetherin (Fig. 1c). We assume that its lack of activity is explained by its loss of the Vpu interaction site shown to be in the transmembrane (Gupta *et al.*, 2009a; Kobayashi *et al.*, 2011; Lim *et al.*, 2010; McNatt *et al.*, 2009). Thus, the transmembrane domain is not required for TGN localization but is required to recruit tetherin. Together these results suggest that Vpu needs to be localized to the TGN in order to rescue virion release, but that it also needs the M group transmembrane region to be able to recruit tetherin. Importantly, in all cases except the transmembrane swap, which cannot recruit tetherin (Kobayashi *et al.*, 2011; McNatt *et al.*, 2009), the proteins that localize to the TGN have robust anti-tetherin activity in the virus release assay. The amount of colocalization quantified by determining the PCC for each Vpu construct reflected the images shown.

**Fig. 4.**
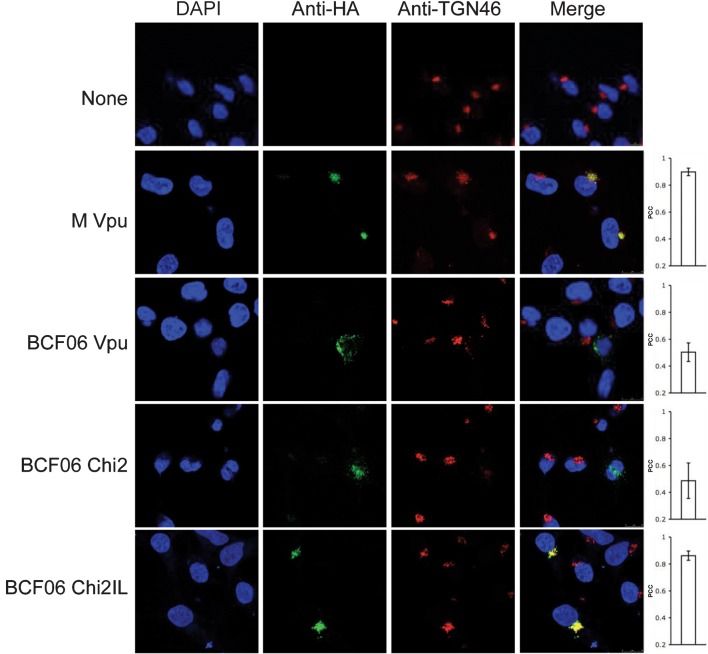
O group Vpu disrupts the Golgi network. Confocal images of 293T cells cotransfected with the various Vpu constructs and stained with immunofluorescently labelled anti-HA (Alexa-488) and anti-TGN46 (rhodamine). Cell nucleus is detected with DAPI and merged images are in the fourth column. Each image is representative of at least four different fields positive for each HA-construct, taken at random. The PCC was calculated for each HA-construct. Values represent mean±sem.

Surprisingly, BCF06 O group Vpu, as well as all the chimeras unable to antagonize human tetherin, appeared to disrupt the TGN marker p230 (Golgin) (Fig. 3). Indeed, expression of these Vpus shifted this marker from a typical punctate TGN localization to a much more dispersed staining pattern. Cells in the same field that did not express Vpu showed a normal punctate TGN signal. To test whether this effect was specific to the p230 protein, we repeated the experiment with anti-TGN46 antibodies (Fig. 4). The same result was observed for this antigen, suggesting that O group Vpu and the chimeras that cannot antagonize tetherin disrupt the TGN. Quantification of the colocalization also correlated with these results. BCF06 (PCC = 0.50) and BCF06 chimera 2 (PCC = 0.49), neither of which antagonize tetherin, showed diffuse localization appearing to disrupt the TGN. NL4-3 Vpu (PCC = 0.90) and BCF06 chimera 2IL (PCC = 0.86), which antagonize tetherin, showed a typical punctate colocalization of TGN46 and Vpu. TGN disruption by Vpu has been observed previously (Hauser *et al.*, 2010); however, the mechanism of this disruption and the cellular consequences of this remain unclear.

Finally, we tested whether O and M group Vpus colocalized with human tetherin. 293T cells cotransfected with O and M group HA-tagged Vpus and human tetherin were stained for HA and tetherin (Fig. 5). Tetherin expressed alone was dispersed throughout the cell in a vesicular pattern as well as on the plasma membrane as described previously (Gupta *et al.*, 2009b; Kupzig *et al.*, 2003; Neil *et al.*, 2008; Van Damme *et al.*, 2008). Co-expressing tetherin with M group Vpu (PCC = 0.72) or BCF06 chimera 2IL (PCC = 0.71), which antagonize tetherin, recruited it to a punctate location, most probably the TGN (Fig. 3), where they are entirely colocalized. BCF06 O group Vpu (PCC = 0.40) did not relocalize tetherin, and the tetherin remained dispersed throughout the cell. BCF06 chimera 1IL (PCC = 0.55) also did not bring tetherin to a punctate location, concordant with its inability to antagonize tetherin function (Fig. 1). Therefore, colocalization quantification results suggest that the association of Vpu with the TGN, as well as the recruitment of tetherin by Vpu to a punctate localization, are essential for Vpus’ enhancement of virion release. O group Vpus are defective in this function and this appears to correspond with an ability to disrupt the localization of Golgi proteins and perhaps the TGN itself.

## Discussion

In this study, we have shown that the O group Vpu protein is unable to antagonize tetherin due to sequences in its transmembrane and membrane proximal regions. Our data are concordant with the observation that sequences in the transmembrane and membrane proximal regions of Vpu influence Vpu’s specificity against species variants of tetherin (Gupta *et al.*, 2009a; McNatt *et al.*, 2009). Our data also suggest that the sequence of the Vpu extracellular N terminus also plays a minor role. Localization of Vpu to the TGN and the pericentriolar recycling endosomes has been shown to be important for its ability to enhance virion release (Varthakavi *et al.*, 2006). For HIV-1, M group viruses from subtypes B and C, it was shown that the Vpu transmembrane proximal region motifs YXXφ and (D/E)XXXL(I/L) are responsible for this localization (Dubé *et al.*, 2009; Ruiz *et al.*, 2008). Here, we show that HIV-1 M group localizes to the TGN, whereas the O group Vpu does not. Furthermore, we show that this is dependent on isoleucine and leucine residues, within the YXXφ motif. Incorporating these residues into the O group Vpu moves the protein to the TGN where it colocalizes with both p230 Golgin and TGN46. Whether this motif is a true sorting signal remains unclear as its mutation could simply alter the conformation of the Vpu cytoplasmic tail and then affect cofactor interactions. Chimeras that also include the M group transmembrane region, and can therefore bind tetherin, as well as the M group N-terminal domain, induce TGN localization and additionally bestow anti-tetherin function. Active anti-tetherin chimeras were able to recruit human tetherin to a punctate location, most probably the TGN, whereas O group Vpus and the inactive O/M chimeras could not. Our observations therefore support the model that relocalization of tetherin to the TGN, away from the site of budding, is important for tetherin antagonism and the enhancement of virion release by Vpu. BCF06 chimera 1IL appears to remove tetherin from the cell surface, but cannot antagonize tetherin nor relocalize it to a punctate, TGN-like location. This mutant suggests that removal of tetherin from the cell surface may not be sufficient for antagonism and some intracellular interaction between tetherin and virus may still occur. Our demonstration that tetherin antagonism is dependent on the sequence of the transmembrane region is concordant with reported observations. For example, it was recently shown that the determinants of Vpu within its transmembrane domain are residues 1–8 and 14–22 (Lim *et al.*, 2010), with SIVcpz Vpu being able to rescue restriction by human tetherin when it included both of these regions from HIV-1 M group Vpu. Furthermore, the tetherin transmembrane domain has been shown to be highly positively selected and encode the species-specific determinants of sensitivity to antagonism by Vpu (Gupta *et al.*, 2009a; McNatt *et al.*, 2009). Thus, in order to sequester tetherin in the TGN, Vpu must both localize to the TGN and also be able to recruit tetherin by virtue of its transmembrane domain. Concordantly, M group Vpu bearing an O group transmembrane region M [OTM] was localized appropriately to the TGN, but could not antagonize tetherin presumably due to the loss of its tetherin-binding site. Our data suggest that O group viruses have failed to adapt their Vpu molecules to either interact with tetherin or localize to the TGN.

It is interesting to note that O group Vpu and chimeras unable to locate to the TGN disrupted the tight punctate perinuclear staining of two independent TGN markers p230 Golgin and TGN46. TGN distortion by Vpu has previously been observed (Hauser *et al.*, 2010; Van Damme *et al.*, 2008). Similar TGN disruption has been demonstrated for other proteins, such as dynamin and Hip1R (Cao *et al.*, 2000; Carreno *et al.*, 2004). O group Vpus from Ca9 and BCF06 are thus able to disrupt the TGN, which is likely to impact on cell function and viability, although the implications of this observation during natural infection remain unclear. Importantly, it is possible that the TGN marker redistribution reflects specific redistribution of the markers rather than disruption of the whole organelle.

Both O and M group Vpu molecules are able to remove CD4 from the cell membrane. However, the failure of most of the O/M chimeric Vpus to act on CD4 suggests that the interaction between CD4 and the different Vpus are complex, involving different molecular interactions between O and M Vpus and CD4. Anti-tetherin activity and anti-CD4 activity are likely mechanistically distinct, although we note that in the main, the chimeras that were able to remove CD4 from the surface were also able to remove tetherin from the surface, compare plots Fig. 2(a) and Fig. 2(b). This suggests some similarity between the mechanisms of anti-CD4 and anti-tetherin activity and we speculate that Vpu may prevent surface localization of tetherin rather than remove it from the cell surface as it does for CD4. There are, however, differences between tetherin and CD4 manipulation as evidenced by the O group Vpus ability to antagonize CD4 but not tetherin. Furthermore, BCF06 Chi3, which encodes amino acids 1–40 of the M group Vpu, efficiently removes tetherin from the cell surface but has a defect in removing CD4. This appears to be somehow due to the N terminus as replacing the M group sequence with the O group N terminus to form Chi4 restores anti-CD4 activity, whilst maintaining anti-tetherin activity (Fig. 2). Our observations suggest complex relationships between domains of Vpu, particularly the N terminus and membrane proximal region, which are either side of the plasma membrane. Importantly, all the chimeras able to antagonize tetherin, as measured by a viral release assay (Fig. 1), effectively remove tetherin from the cell surface. The results of immunohistochemistry are also concordant with tetherin surface staining by flow cytometry.

Together with the study from Kirchhoff and colleagues (Sauter *et al.*, 2009) our data demonstrate how O group Vpu has failed to acquire the anti-tetherin activity acquired by M group HIV-1 during its adaptation to humans. The difficulty of making this adaptation is suggested by the complex multi-functional nature of Vpu and the obvious difficulty of adapting the protein to new roles without impacting on its ability to perform its original functions. This is most vividly illustrated by the observation that whilst both O and M group Vpus effectively remove CD4 from the cell surface, most of the O/M chimeric Vpus did not (Fig. 2). HIV-1’s adaptation to antagonize tetherin suggests that tetherin has been an important component of the species barrier between chimpanzees and humans. Whilst M group HIV-1 has made this adaptation, O group virus has not. There are far fewer O group infections than M group infections and all O group infections can be directly linked to Africa (Gao *et al.*, 1999). However, the lower number of O group infections cannot be explained by a more recent zoonosis as compared to M group (Lemey *et al.*, 2004). Furthermore, whilst O group HIV-1 is unable to counteract tetherin, it is still able to transmit between individuals and cause disease. It is therefore an attractive theory that tetherin’s ability to prevent the release of O group HIV-1 contributes to the reduced spread that must underlie the lower number of infected individuals. We expect that the continued consideration of how non-pandemic O group virus differs from pandemic M group virus is likely to be informative when considering preventative strategies including protective vaccines.

## Methods

### 

#### Molecular cloning.

Vpu sequences were obtained by PCR amplification of DNA from human T-cells infected with Ca9 and BCF06 viral isolates (Janssens *et al.*, 1999; Loussert-Ajaka *et al.*, 1995). Constructs were cloned into a C-terminally HA-tagged pcDNA3. Ca9 Chi1, M [OTM] and BCF06 Chi1 were synthesized by Genscript and then cloned into HA-tagged pcDNA3. Other chimeras were generated by site-directed mutagenesis. For CD4 and tetherin cell surface downregulation assays, the Vpu constructs were cloned into pIRESeGFP (Clontech), with and without an HA-tag. (All primer sequences are available on request.) GenBank accession number for BCF06 HIV-1 Vpu is AB485666 and the submitted Ca9 Vpu sequence is HQ857213.

#### Viral infection assays.

Preparation of VSV-G pseudotyped, YFP encoding HIV-1 has been described previously (Gupta *et al.*, 2009b). Untagged human tetherin construct (100 ng) was co-transfected along with HIV-1 vector plasmids and titrations of the various Vpu expressing plasmids or empty vector (pcDNA3.1; Invitrogen) ranging from 50 to 1000 ng. After 48 h the supernatant was harvested, filtered and titrated onto naïve 293T cells.

#### Western blot analysis.

HIV-1 p24 was measured in supernatants or cell pellets by Western blot analysis as described previously (Gupta *et al.*, 2009b). Membranes were stripped and reprobed for β-actin (Abcam) as a loading control and HA (Covance). Cells were lysed in RIPA buffer. Cleared lysates or supernatant were added to Laemmli buffer and the samples were then boiled before separation by SDS-PAGE, as described previously (Gupta *et al.*, 2009b).

#### Immunofluorescence.

HEK293T cells on poly-l-lysine coated coverslips were transfected with 500 ng BCF06 Vpu or its chimeras using Fugene 6. Cells were then fixed in 3 % PFA and permeabilized in 0.1 % Triton X-100 and stained with anti-HA (Roche), anti-tetherin (Abnova), anti-TGN46 (Serotec) or anti-p230 *trans*-Golgi antibodies and appropriate secondary antibodies. Colocalization was quantified using ImageJ software (http://rsb.info.nih.gov/ij/). The PCC was calculated for four separate fields positive for each HA-construct, taken at random, by using the Mander’s Coefficient plugin from the ImageJ Colocalization Analysis package (Dunn *et al.*, 2011; Manders *et al.*, 1993).

#### Cell surface removal assay.

HEK293T cells were transfected as above with 300 ng of a CD4, or 400 ng of an ectodomain HA-tagged human tetherin expression vector and 1 µg pIRES2-eGFP (Vpu) plasmid. Post-transfection (48 h), cell surface CD4 and tetherin expression were examined by flow cytometry after staining with a CD4 antibody (Serotec), or with an anti-HA antibody (Covance). The mean fluorescence intensity for CD4 or tetherin expression was compared, after gating cells expressing GFP, as described previously (Sauter *et al.*, 2009).
